# Repeated sessions of PACK-CXL WA for the treatment of resistant bacterial keratitis: a retrospective study

**DOI:** 10.1186/s12886-023-03080-3

**Published:** 2023-07-26

**Authors:** Mohammed M. Mahdy Tawfeek, Hanan Mohamed Abdel Hamid Ahmed, Ashraf Bor’i, Ahmed M. Nashaat Ali Rady

**Affiliations:** 1 Zagazig University, MRCSEd, MRCS (Ophth.) (Glasg.), FICO, Zagazig, Egypt; 2 General Organization Of Teaching Hospitals and Institutes (GOTHI), Mataria Teaching Hospital, Cairo Governorate, Egypt; 3 Zagazig University, Zagazig, Egypt; 4 Merit University, MRCSEd, Sohag Governorate, Egypt

**Keywords:** Corneal cross linking, PACK-CXL, Bacterial keratitis, Window absorption, Repeated

## Abstract

**Objective:**

The aim of this work is to evaluate the safety and efficacy of repeated sessions of photo-activated chromophore for keratitis-cross linking (PACK-CXL) window absorption (WA) for the treatment of resistant bacterial keratitis (BK).

**Patients and methods:**

This is a retrospective clinical cohort study. Thirty eyes with clinically suspected and lab-confirmed bacterial keratitis, resistant to appropriate antibiotic therapy- which was modified by sensitivity reports- for 2 weeks with failure of epithelialization for 4 weeks after the standard anti-microbial therapy (SAT) together with one setting of PACK-CXL WA were included. If after the first session of PACK-CXL, there is a start of improvement in the form of reduction of the size of corneal ulcer and stromal infiltrates together with the start of epithelialization on clinical examination and AS-OCT, another session of PACK-CXL WA was performed after one week, and so on, till the complete healing and resolution of bacterial keratitis and confirmation by negative bacterial culture. Identification of the micro-organisms was done by lab study before and after treatment. Corneal healing was evaluated by corneal examination and anterior segment OCT (AS-OCT).

**Results:**

Thirty eyes of 30 patients were recruited in this study. They were 16 males and 14 females, their mean age was 44.3 ± 5.38 years. The mean ulcer size was 3.96 ± 1.87 (mm^3^), while the mean size of stromal infiltrates was 4.52 ± 2.24 (mm3). PACK-CXL WA treatment was performed an average of 2.87 times for the 30 eyes. Complete healing and resolution (Successful treatment) was observed in 27 eyes (90%) of cases and failure of epithelialization was observed only in 3 eyes (10%). Complete corneal healing was reported in the second month postoperatively in 90% of eyes.

**Conclusion and recommendation:**

PACK-CXL WA may be a promising, non-invasive treatment option for resistant bacterial keratitis. It may have a synergistic effect with standard antimicrobial treatment (SAT). Also, it can overcome the antibiotics resistance that has become rapidly spreading worldwide. Repeated sessions of PACK-CXL WA may be more effective for the treatment of resistant bacterial keratitis till complete epithelialization and resolution of BK than a single session with few complications. However, further prospective and comparative studies to support the results are needed.

## Introduction

Infectious keratitis represents one of the leading causes of blindness worldwide. It can result from minor ocular trauma because of occupational risk [1, 2] combined with poor hygiene. Particularly, in the absence of immediate and adequate medical care - which is often in developing countries- it is a sight-threatening condition. Although the incidence rate in developed countries is significantly lower, infectious keratitis can arise from recent ocular surgery, contact lens wear, [3] dry eyes, [4] or systemic immunosuppression [5, 6].

The multiplicity of the underlying microorganisms- including bacteria, fungi, and protozoa—makes clinical diagnosis [7] and hence adequate antimicrobial treatment challenging, especially because laboratory analyses are not immediately available. In the absence of adequate antibiotics treatment, infectious ulcers can progress rapidly [8].

A further concern in the management of infectious keratitis is multi-resistant bacterial strains [9, 10]. The standard bacterial keratitis treatment according to the guidelines of the American Academy of Ophthalmology includes the use of broad-spectrum topical antibiotics. However, antimicrobial resistance (AMR) is rising at an alarming speed, and the World Health Organization (WHO) has published an urgent call to identify alternatives to antibiotics in its global report on AMR [2].

If the first-line antibiotic medication is not effective, halting the ulcer progression is delayed. The antimicrobial effect of riboflavin photo-activation has been explained for potential applications in treating infectious keratitis [11].

The antibacterial properties of CXL are a result of UV light’s interaction with the chromophore riboflavin. It renders pathogens, such as bacteria and viruses, inert by damaging both their DNA and RNA [12].

Riboflavin also absorbs photons and produces reactive oxygen species (ROS). These free radicals form new covalent bonds by connecting particular amino acids in nearby collagen fibres and proteoglycan molecules [13].

The center of the ulcer was gently swiped to remove all cellular debris without completely de-epithelizing the cornea, creating a window for hyp-osmolar riboflavin installation (absorption) and radiation in a novel procedure known as corneal cross-linking window absorption (CXL-WA) for the treatment of keratitis as described by **Rosetta el al. (2013)** [14].

The purpose of this work is to evaluate the safety and efficacy of repeated sessions of PACK-CXL WA for the treatment of resistant bacterial keratitis.

## Patients and methods

This retrospective cohort interventional study was conducted on 30 eyes of adult patients with clinically suspected and lab-confirmed bacterial keratitis which did not respond to appropriate standard antimicrobial therapy (SAT) which was modified according to sensitivity reports for 2 weeks. All these eyes underwent one session of PACK-CXL WA 2 weeks after presentation without response to SAT. If after the first session of PACK-CXL, there is a start of improvement in the form of reduction of ulcer size and stromal infiltrates size together with start of epithelialization on clinical examination and AS-OCT, another session of PACK-CXL WA was performed after one week, and so on, till the complete healing and resolution of bacterial keratitis and confirmation by negative bacterial culture. Repeated sessions of PACK-CXL was performed 1 week apart for maximum of 4 sessions till complete healing after 2 months or failure of treatment. Four repeated sessions were used as a maximum as the central corneal thickness was found to be just adequate of more than 400 μm with epithelium as measured by anterior segment OCT (AS-OCT) before performing the 4th setting in all cases.

Patients were collected from El-Tayseer Eye Hospital in Zagazig, Egypt, in the period from May 2022 to November 2022 where all the surgical procedures were carried out. This study was approved by the research ethical committee and the Institutional Review Board (IRB) of the Faculty of Medicine, Zagazig University, Egypt and met the ethical code of the World Medical Association for human experimentation, as stated in the Helsinki Declaration. Written informed consent was obtained from all study participants. In non-educated patients, the written informed consent was taken from the parents of the patients or legal relatives.

The patients’ age was equal to or over 18 years, and both sexes were included in the study, also central corneal thickness (CCT) of more than 400 μm with epithelium as measured by anterior segment OCT (AS-OCT) together with Infiltrates involving less than 250 μm depth of corneal thickness (up to mid-stromal level) and/or safety zone above the corneal endothelium without infiltrations of more than 100 μm as measured by Anterior segment OCT (AS-OCT) were included. However, patients who had perforated corneal ulcers or impending corneal perforation, scleral involvement, total corneal or corneal involvement near to limbus by 1 mm, endophthalmitis, viral or fungal or acanthamoeba or mixed keratitis, and non-infectious keratitis were excluded. Also, known allergy to study medications; Pregnancy or lactation were excluded.

### Preoperative measures

The growth on culture media was used to form a film stained with different stains for visualization of the different types of bacteria microscopically, called direct smears.

The size of the ulcer and abscess was estimated at the slit-lamp evaluation in three dimensions (mm^3^) and with anterior segment optical coherence tomography (AS-OCT; RTVue Optovue Inc., Fremont, CA) measurement.

Anti-microbial protocol was Moxifloxacin 0.3% (Vigamox eye drop; Alcon Laboratories Inc.) every 3 h. Treatment was modified according to results of culture and sensitivity.

### Technique of PACK-CXL WA

The CXL-WA treatment was performed under topical anesthesia with benoxinate hydrochloride 0.2% (Benox eye drops) administered 2 min before surgery.

The procedure was conducted in sterile surgical conditions. The patient was draped and a lid speculum was applied.

A hypo-osmolar 0.1% riboflavin solution (RICROLIN TE Sooft, Italy) was instilled for 30 min before irradiation, to obtain stromal swelling. The cornea was exposed to ultraviolet light A with the UV-X System (Peschke Meditrade GmbH, Huenenberg, Switzerland), which emits light at a wavelength of l370 ± 5 nm and an irradiance of 3 mW/cm^2^ or 5.4 J/cm^2^. Exposure lasted for 30 min, during which time riboflavin solution was applied 15 times, every 2 min.

After surgery, the patient received standard antimicrobial therapy (SAT) - modified according to sensitivity reports - together with supplementary therapy for infectious keratitis as cycloplegic eye drops, tear substitutes and topical ocular hypotensives if the intra-ocular pressure (IOP) is digitally high.

Successful treatment was defined as there is a start of improvement in the form of reduction of ulcer size and stromal infiltrates size together with start of epithelialization till complete healing and resolution of BK with corneal scar formation together with negative bacterial culture. However, failure of treatment was defined as a failure of appearance of any signs of improvement inspite of four maximum repeated sessions of PACK-CXL with persistent epithelial defects, and other treatment options as amniotic membrane graft (AMG) was then performed.

### Follow-up

Patients were evaluated daily by slit lamp for 1 week, every week for 1 month and every month for 2 months. Clinical photos using both photo-slit lamp and AS-OCT (AS-OCT; RTVue Optovue Inc., Fremont, CA), were taken before and after treatment. Follow up visits included reporting signs of healing as diminished ulcer size and stromal infiltrates and abscess size up to complete resolution; healing of epithelial defects together with corneal scarring and vascularization with negative bacterial cultures.

### Statistics

The collected data were coded, entered, presented and analyzed by computer using a data base software program, Statistical Package for Social Science (SPSS) version 20. Mean ± SD, chi-square and t-test were used for determination of significance (P value). P < 0.05 is considered significant.

## Results

Thirty eyes of 30 patients were recruited in this study. They were 16 males and 14 females, their mean age was 42.7 ± 3.56 years. They were 14 educated and 16 ignorant patients. On presentation, visual acuity of > 6/60 was reported in 6 patients, 15 patients had visual acuity of < 6/60 up to counting fingers (CF) and 9 patients had visual acuity of hand movement (HM) (Table 1).

**Table 1:** Patients’ characteristics of the study population

	Total		Males		Females		Significance	
	No.	%	No.	%	No.	%	χ^2^	P value
Patients	30	100	16	53.3	14	46.7	0.009	0.523
Urban	11	36.7	6	20.0	5	16.7	1.649	0.016*
Rural	19	63.3	10	33.3	9	30.0		
Educated	14	46.7	7	23.3	7	23.3	0.012	0.344
Ignorant	16	53.3	9	30.0	7	23.3		
VA > 6/60	6	20.0	3	10.0	3	10.0	0.000	1.000
VA < 6.60 up to CF	15	50.0	9	30.0	6	20.0	0.105	0.096
VA ≤ HM	9	30.0	4	13.3	5	16.7	0.101	0.099
	Mean ± SD		Mean ± SD		Mean ± SD		T	P value
Age (years)	44.3 ± 5.38		44.5 ± 5.42		43.9 ± 5.31		0.003	0.874

χ^2^ = Chi square test, P > 0.05 = non-significant, *p < 0.05 = statistically significant,

VA: visual acuity, HM: hand movement, CF: counting fingers

Twelve eyes (40% of cases) were Gram-positive cocci most of them were Staph. aureus, followed by coagulase-negative Staphylococci (CONS); 10 eyes (33.3%). Three eyes (10%) were Gram-positive bacilli, other organisms were represented in 5 (16.7%) eyes (Table 2).

**Table 2:** Etiology of bacterial keratitis in the studied patients

Type	Species	N = 30	%
Gram-positive cocci	Staphylococcus aureus Staphylococcus epidermidis	12	40.0
Coagulase-negative Staphylococci (CONS)	Staphylococcus	10	33.3
Gram-positive bacilli	Corynebacterium diphtheriae Corynebacterium xerosis	3	10.0
Gram- negative bacilli	Pseudomonas aeruginosa	1	3.33
Enterobacteriaceae species	Escherichia coli	1	3.33
Gram-negative diplococci	Neisseria gonorrhoeae	1	3.33
Gram-negative diplobacillus	Moraxella lacunata	1	3.33
Non-TB mycobacterium	Mycobacterium chelonae	1	3.33

As regard risk factors; 12 eyes (40%) were represented by exogenous local risk factors, while endogenous local factors were represented in 10 eyes (33.3%) and systemic risk factors (Diabetes mellitus) were represented in 8 (26.7%) of eyes (Table 3).

**Table 3:** Risk factors of bacterial keratitis in the studied patients

Type	Details	N = 30	%
Exogenous local factors	- Chronic contact lens wear, topical corticosteroids chronic use, ocular trauma.	12	40.0
Endogenous local factors	- Eyelid disorders (entropion, trichiasis) - Corneal disorders (herpetic eye diseases, and past corneal surgeries including keratoplasty, and keratorefractive surgery).	10	33.3
Systemic risk factors	- Diabetes Mellitus (D.M.)	8	26.7

The mean ulcer size decreased from 3.96 mm^3^ after 2 sessions to 2.18 mm ^3^ and 0.57 mm^3^ after 3 and 4 sessions, respectively. The infiltrate size had a mean of 4.52 mm^3^ after 2 sessions and decreased to 3.16 and 1.93 mm^3^ after 3 and 4 sessions, respectively (Table 4). PACK-CXL WA treatment was performed an average of 2.87 times in the 30 eyes. The response was found to be much better in Gram positive BK (24 eyes) than Gram negative BK (2 eyes) than mycobacterial keratitis (1 eye).

**Table 4:** Size of ulcer and stromal infiltrates

Outcome	2 sessions (n = 30)		3 sessions (n = 18)		4 sessions (n = 8)		F	P
Size of lesion	Mean	±SD	Mean	±SD	Mean	±SD	MW	P
Infiltrate size (mm^3^)	4.52	2.24	3.16	1.75	1.93	1.11	1.273	0.000*
Ulcer size (mm^3^)	3.96	1.87	2.18	1.12	0.57	0.46	1.254	0.000*

F: Fisher exact test, MW: Mann Whitney test

**Fig. 1:**
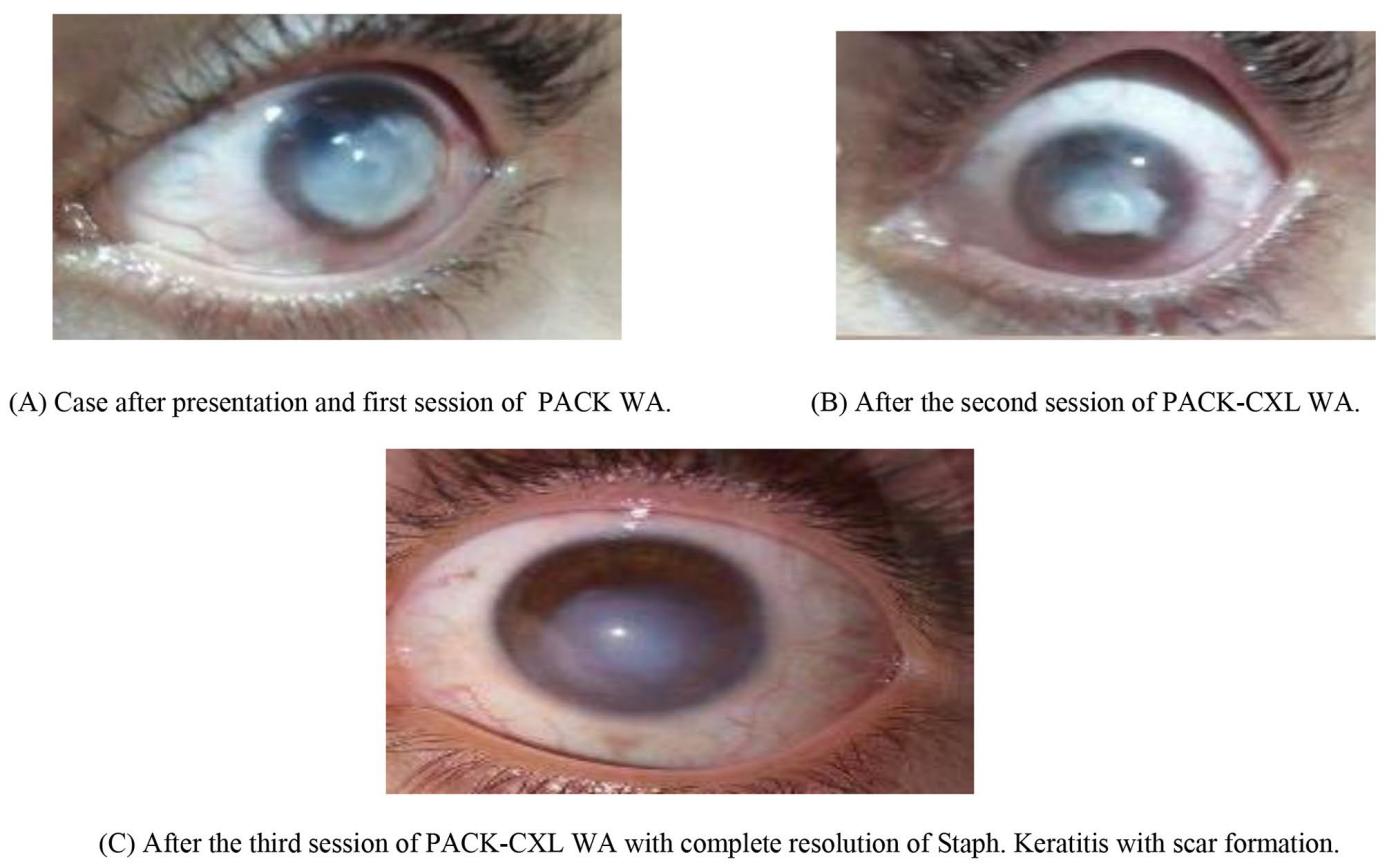
A case of resistant Staph. Keratitis after surface ablation laser surgery treated by 3 repeated sessions of PACK-CXL WA one week apart

Successful treatment was reported in 12 eyes (40%), 10 eyes (33.3%), 5 eyes (16.7%), while failure was observed in 18 eye (60%), 8 eyes (26.7%) and 3 eyes (10%) after 2, 3, (Figs. 1) and 4 sessions, respectively. The overall success rate was 90% after the repetition of 4 sessions of PACK-CXL WA one week apart, while, the overall failure rate was 10% after the repetition of 4 sessions of PACK-CXL WA one week apart (Table 5). In the three eyes which were resistant to four repeated sessions of PACK-CXL WA, with delayed epithelialization, amniotic membrane grafting (AMG) was performed, together with the antibacterial therapy modified by the antibiogram, till complete healing and corneal vascularization was attained.

**Table 5:** Outcome of PACK-CXL WA among study patients according to number of sessions

Outcome	2 sessions (n = 30)			3 sessions (n = 18)		4 sessions (n = 8)			F	P
	No.	%		No.	%	No.		%		
Successful treatment	12	40.0		10	33.3	5		16.7	6.247	0.009*
Treatment failure	18	60.0		8	26.7	3		10.0	7.152	0.008*
Overall success	Success-treated eyes				Failed-treated eyes				Significance	
	No.		%		No.		%		χ^2^	P
Patients (n = 30)	27		90		3		10		24.67	0.003*

F: Fisher exact test

Complications were absent in 25 eyes (83.3%), 27 eyes (90%), and 28 eyes (93.33%) after 2, 3, and 4 sessions, respectively. However, 5 eyes (16.7%), 3 eyes (10%), and 1 eye (3.3%) had impending perforation after 2, 3, and 4 sessions, respectively. Only one eye (3.3%) had perforation after the 4th session (Table 6).

**Table 6:** Complications of PACK-CXL WA among study patients

Complications	2 sessions (n = 30)		3 sessions (n = 18)		4 sessions (n = 8)		F	P
	No.	%	No.	%	No.	%		
• Absent	25	83.3	27	90	28	93.3	0.312	0.435
• Impending perforation	5	16.7	3	10	1	3.33	1.174	0.129
• Perforation	0	0.00	0	0.00	1	3.33	2.963	0.085

F: Fisher exact test, P > 0.05 statistically insignificant

## Discussion

Photo-Activated Chromophore for Keratitis window absorption (PACK-CXL WA) is now a promising agent for treatment of infective keratitis especially in resistant cases. Authors reported using of such treatment modality for advanced corneal melting in treatment-resistant cases of infectious keratitis [15, 17] alone or recently in combination with medical or surgical treatment [18, 20]. These studies chose patients unresponsive to standard topical and systemic antimicrobial therapy and prompted the use of CXL as a rescue measure, then observed the outcome of these patients [16]. Previous results [13] confirmed such treatment modality and proposed that CXL may be effective for the treatment of infectious corneal erosions. Several case series, two randomized controlled trials [15, 21], a systematic review [22] and one meta-analysis [22] showed similar findings. The postulated mechanisms that may illustrate the role of repeated sessions of PACK-CXL WA for treating resistant cases of bacterial keratitis may be summarized in two main actions: Bacterial eradication by damaging bacterial DNA and RNA (especially of the antibacterial-resistant strains), together with, enhancement of epithelial healing through stromal collagen enhancement and Bowman’s membrane stabilization.

Causative organisms of infectious keratitis found in our study were similar to those reported in the literature with bacterial infection [24]. Most of them was Gram-positive cocci, mainly *Staph*. *aureus*, found in 40% of eyes followed by coagulase-negative cocci in 33.3% of eyes, 10% were Gram-positive bacilli, other organisms represented in 16.7% of eyes.

The risk factors in the current study were mostly exogenous local risk factors (40%), mostly due to contact lens abuse, endogenous local factors represented one third (33.3%) and systemic risk factors represent 26.7% of eyes. This was agreed by other literatures [1] in which contact lens and other exogenous factors was responsible on most of bacterial keratitis.

We have only 3 cases failed (10%) at the final session while 90% of cases had successful treatment at final follow-up.

Similarly, **Papaioannou et al.** [23] reported a healing rate of 87.2% (159 of 175 eyes) in a systematic review and meta-analysis of 2 randomized controlled clinical trials, 13 case series, and 10 case reports. Also, **Gulias-Cañizo et al.** [19] found a similar rate (90.5%) as our study. This finding is consistent with other studies [17, 21] where patients with bacterial keratitis show improvement after PACK-CXL treatment. PACK-CXL WA treatment was performed an average of 2.87 times for the 30 eyes. However, these studies only used a single session of PACK-CXL.

**Gulias-Cañizo et al.** [19] highlighted that between day 1 and week 1 after treatment, the ulcer size does not decrease or even increases slightly compared to baseline and then decreases after week 1. This should not be a reason for considering that the treatment is not successful, since this response is due to the mechanical de-epithelialization of the ulcer margins for CXL application and not a sign of deterioration. The slow healing rate in the first week (consistent with re-epithelialization) contrasts with the strong and sustained response to treatment showed by the decreased ulcer size and the healing response after this time-point, reaching a maximum at 3 months.

The healing rates obtained with PACK-CXL are unprecedented positive results for those patients, hence the importance of sharing our results to prompt a wider use of this procedure in cases unresponsive to standard therapy. Microbial resistance to antibiotics increases, so, new lines of treatment are needed. PACK-CXL may be a promising new alternative and its use is recommended due to the potential benefit obtained by controlling infection regardless of drug resistance, stopping the melting process, avoiding emergency keratoplasty, decreasing the possibility of performing lamellar grafts for visual rehabilitations and decreasing rate of corneal graft rejection due to its role in suppressing corneal vascularization.

## Conclusion and recommendation

PACK-CXL WA may be a promising, non-invasive treatment option for resistant bacterial keratitis. It may have a synergistic effect with standard antimicrobial treatment (SAT). Also, it can overcome the antibiotics resistance that has become rapidly spreading worldwide. Repeated sessions of PACK-CXL WA may be more effective for the treatment of resistant bacterial keratitis till complete epithelialization and resolution of BK than single session with little complications. However, further prospective and comparative studies to support the results are needed.

## Data Availability

The data-sets generated and analyzed during the current study are not available due to the protection of data security (the original data contains a lot of specifically demographic characteristics information and will be used again in the future follow-up study) but are available from the corresponding author on reasonable request.
